# Assessment of respiratory effort with EMG extracted from ECG recordings during prolonged breath holds: Insights into obstructive apnea and extreme physiology

**DOI:** 10.14814/phy2.14873

**Published:** 2021-05-27

**Authors:** Mark Stewart, Anthony R. Bain

**Affiliations:** ^1^ Department of Physiology & Pharmacology SUNY Downstate Health Sciences University Brooklyn NY USA; ^2^ Department of Kinesiology Faculty of Human Kinetics University of Windsor Windsor ON Canada

**Keywords:** breath‐holding, dry apnea, free diving, sleep apnea

## Abstract

Breath holding divers display extraordinary voluntary control over involuntary reactions during apneic episodes. After an initial easy phase to the breath hold, this voluntary control is applied against the increasing involuntary effort to inspire. We quantified an electromyographic (EMG) signal associated with respiratory movements derived from broad bandpass ECG recordings taken from experienced breath holding divers during prolonged dry breath holds. We sought to define their relationship to involuntary respiratory movements and compare these signals with what is known to occur in obstructive sleep apnea (OSA) and epileptic seizures. ECG and inductance plethysmography records from 14 competitive apneists (1 female) were analyzed. ECG records were analyzed for intervals and the EMG signal was extracted from a re‐filtered version of the original broad bandpass signal and ultimately enveloped with a Hilbert transform. EMG burst magnitude, quantified as an area measure, increased over the course of the struggle phase, correlated with inductance plethysmography measures, and corresponded to significant variance in heart rate variability. We conclude that an EMG signal extracted from the ECG can complement plethysmography during breath holds and may help quantify involuntary effort, as reported previously for obstructive sleep apnea. Further, given the resemblance between cardiac and respiratory features of the breath hold struggle phase to obstructive apnea that can occur during sleep or in association with epileptic seizure activity, the struggle phase may be a useful simulation of obstructive apnea for controlled experimentation that can help clarify aspects of acute and chronic apnea‐associated physiology.

## Graphical abstract

Breath holding divers display extraordinary voluntary control over involuntary reactions during apneic episodes. We show that an EMG signal extracted from the ECG can complement plethysmography as a measure of breath hold respiratory function and may help quantify involuntary effort, as reported previously for obstructive sleep apnea. Further, given the resemblance between cardiac and respiratory features of the breath hold struggle phase to obstructive apnea that can occur during sleep or in association with epileptic seizure activity, the struggle phase may be a useful simulation of obstructive apnea for controlled experimentation that can help clarify aspects of acute and chronic apnea‐associated physiology.

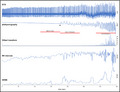

## 1 INTRODUCTION

Extended breath holds by divers experienced with commercial (sustenance diving, e.g., the Ama divers of Japan), recreational (e.g., spear fishing, videography), or competitive (static, dynamic, and depth disciplines) apneic activities have shed important light on cardiorespiratory physiology. The breath hold, which can last many minutes, is comprised of an early quiet or easy phase and a later struggle phase. The prolonged breath hold is one of the best examples of voluntary control over involuntary physiology and breath hold phases have been studied to identify contributors to the diver's voluntary or involuntary decision to break the breath hold (e.gBain et al., 2018; Costalat et al., 2013; Courteix et al., 1993; Cross et al., 2013; Delapille et al., 2001; Guaraldi et al., 2009; Hill, 1973; Honda et al., 1981; Laurino et al., 2012; Parkes, 2006; Schneeberger et al., 1986; Willie et al., 2015).

It is tempting to compare the struggle phase of volitional apnea with clinical apnea, such as occurs in obstructive sleep apnea (OSA). Severe OSA is defined by ≥30 apneic or hypopneic events per hour (AASM, 1999) and individual events can range from 10 seconds (the minimum duration to be counted) to over 60 seconds (Leppanen et al., 2016). The struggle phase in volitional apnea and obstructive apnea is generally defined with inductance plethysmography (e.g., (Kogan et al., 2016)), however, this technique indirectly assesses respiratory effort. Berry and colleagues demonstrated chest wall electromyographic (EMG) signals associated with the electrocardiogram (ECG) were useful in defining periods of obstructive sleep apnea (Berry et al., ,,^2016^, ^2018^, ^2019^). Similar EMG signals associated with periods obstructive apnea during epileptic seizure activity in patients or animal models correlated with inductance plethysmography or intratracheal pressure recordings (e.gLacuey et al., 2018; Nakase et al., 2016; Stewart et al., 2017). Importantly, while inductance plethysmography may be more sensitive to changes in thoracic volume, the EMG signal may provide more meaningful information for actual muscular effort.

Accordingly, we sought to quantify the EMG events occurring in the struggle phase of a breath hold in elite apneists, to define their relationship to involuntary respiratory movements, and compare these signals with what is known to occur in OSA and epileptic seizures. Validation of the inspiratory effort‐associated EMG signal under different clinical apneic conditions also establishes its use in settings where plethysmography is not available.

## 2 METHODS

### 2.1 Divers

Recordings from fourteen competitive apneists (1 female; min 26 years; max 48 years) were analyzed. Average years of competition was 4 years (min 1.5 years; max 14.0 years).

Divers performed breath holds as part of a previous IRB‐approved study with a separate a priori question where subject specifications are detailed (Bain et al., 2015). One breath hold and a preceding “rest” period were analyzed from each diver. The breath hold was performed at total lung capacity, but with no prior hyperventilation.

### 2.2 Recordings

ECG and inductance plethysmography records from each diver were analyzed. ECG recordings were taken from lead II and integrated into a Bio Amp for hardware filtering (AD Instruments). A low‐pass filter was applied with auto adjusted transition width, which was sampled at 1 kHz using LabChart7 Pro (AD Instruments, Colorado Springs, CO, USA). Fast Fourier transform analysis of recorded signals showed that high frequency components existed in the recorded data out to nearly 500 Hz. Inductance plethysmography was performed using a calibrated custom‐built strain gauge strap placed around the midline of the chest and integrated into LabChart7 Pro.

### 2.3 Signal processing

EMG associated with respiratory effort has been shown to mix with the ECG or EEG (Stewart et al., 2017). The EMG signal is higher frequency than most of the ECG or EEG and this can be “extracted” from either signal by high‐pass filtering of an original signal that was collected with a sufficiently wide bandpass. EMG extracted from the ECG represents primarily the thoracic musculature involved in respiration, but “far field” recordings of diaphragmatic EMG are certainly possible and these signals would add with the thoracic EMG.

The ECG, which had originally been recorded with an AC‐coupled wide bandpass, was re‐filtered with a second‐order Butterworth high‐pass filter (>250 Hz), which eliminated essentially all of the ECG signal in most cases. The resulting filtered signal was squared as a method to full‐wave rectify the signal and enhance the signal‐to‐noise ratio, smoothed (50 sample half width), and then enveloped with a Hilbert transform (Balan et al., 2013; Benitez et al., 2001) using National Instruments LabVIEW software (2019, Austin, TX, USA). Peaks in the transform as a function of time were integrated as a magnitude measure (an alternative method is given in (Berry et al., 2019). Units for the resulting Hilbert area measure were filtered squared voltage • seconds. The original ECG signal amplitude and the fraction of the signal passed by the filtering steps made the units potentially difficult to compare across individuals. In 12/14 subjects, the maximum Hilbert areas were similar. The maximum Hilbert area differed from the majority by 10‐fold in one diver and by 100‐fold in another. In Figure 3c, these two subjects were excluded from the plot. The correlation of Hilbert area with plethysmogram features was, however, independent of individual Hilbert area ranges, and was used for comparing all subjects in statistical tests.

RR intervals were determined by finding QRS peaks and calculating the interval between successive R peaks and plotting those differences against the interval midpoints. This is the standard method for evaluating heart rate variability (DeGiorgio et al., 2010; Malik, 1996; Reed et al., 2005). The standard deviation of the RR intervals (SDNN) was computed from 8 successive intervals and plotted against the midpoint of the set of intervals.

### 2.4 Statistics

Plots were constructed with National Instruments LabVIEW (2019; Austin, TX) Microsoft Excel (2016; Redmond, WA, USA), and Graph Pad Prism software (8.4.3; San Diego, CA, USA). Means are plotted with 95% confidence intervals and comparisons across dive phases were made with mixed effects or repeated measures analyses of variance with Dunnett's multiple comparisons tests for post hoc corrections. Statistical significance was considered for *p* values <0.05 and significance levels are given in the text.

## 3 RESULTS

### 3.1 Breath holds

A resting breathing recording was taken from each diver (average recording duration: 2.6 ± 0.9 min; range 1.3 to 4.9 min).

A single breath hold from each diver was studied. During a voluntary breath hold, the diver can voluntarily suppress respiratory movements with relatively less effort (the quiet phase), but eventually, strong involuntary attempts to draw a breath must be met with additional voluntary effort to keep from breathing (the struggle phase) (Bain et al., 2018). The average breath hold duration for this group of divers was 310.3 ± 71.6 seconds (5.2 ± 1.2 min; range: 2.8–7.4 min). The average duration of the quiet phase across all divers was 2.5 ± 0.8 min (range: 1.4–4.0 min) and the average struggle phase duration across all divers was 2.7 ± 0.8 min (range: 0.9–4.1 min). The average arterial carbon dioxide and oxygen tension at the end of the breath hold was 53 and 33 mmHg, respectively. Breath holds were preceded in normoxic conditions with only mild hypocapnia from apnea preparations (average starting arterial carbon dioxide tension at 31 mmHg) (Bain et al., 2015).

### 3.2 EMG bursts occur during struggle phase of prolonged breath holds

**FIGURE 1:**
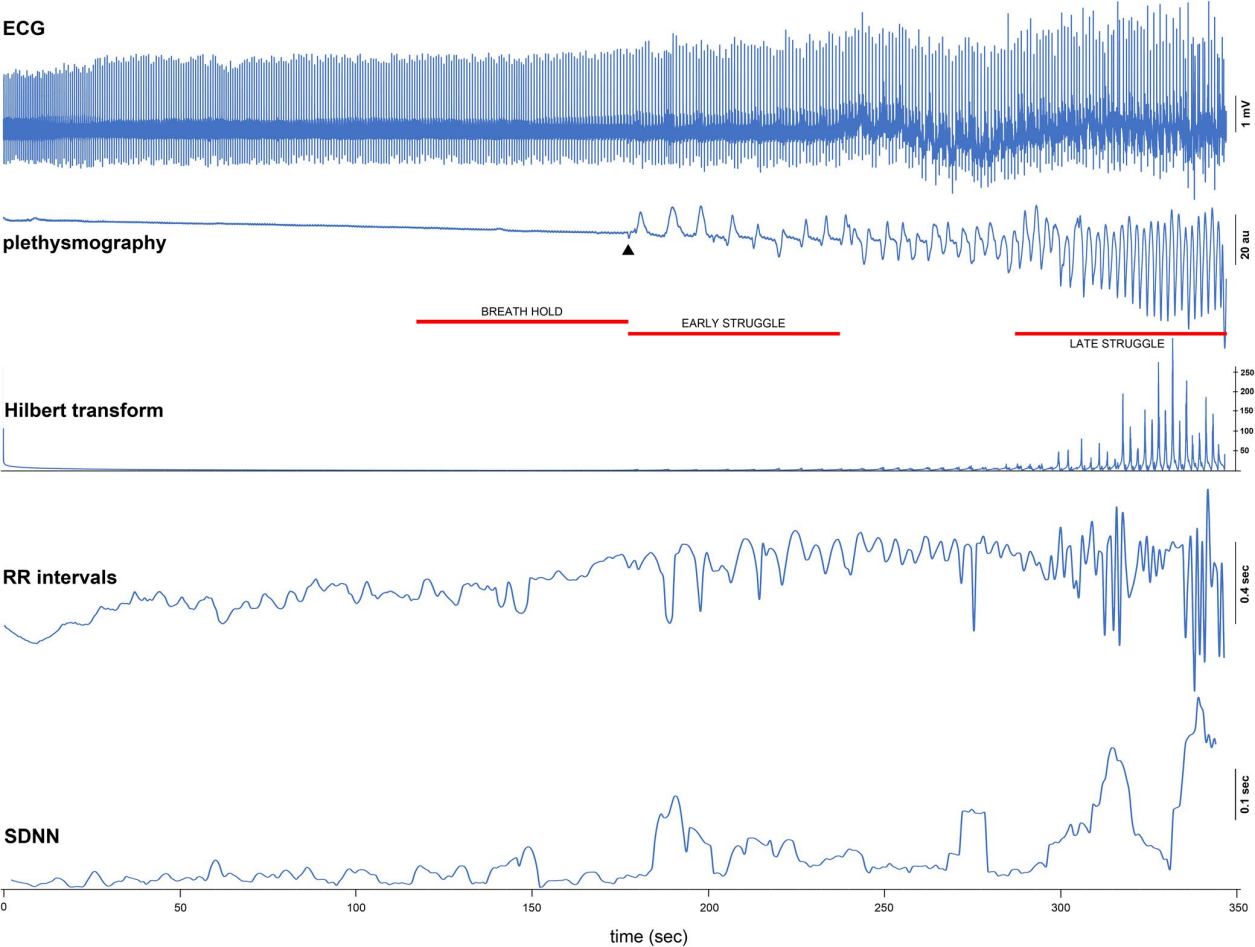
“Raw” and computed metrics from an example breath hold. ECG (top) is shown with the inductance plethysmogram for a breath hold that lasts for 6 minutes. The transition from easy phase to struggle phase is marked by an arrowhead at 178 seconds from the start of the breath hold. The progressive increase in amplitude and frequency of involuntary respiratory attempts is evident (inspiratory effort =upward deflection). Red horizontal lines mark three 60 second periods taken from each breath hold for statistical comparisons. The first, “breath hold,” is a period whose end corresponds to the time of onset of the struggle phase. The second, “early struggle,” is the first 60 seconds of the struggle phase. The last, “late struggle,” is the last 60 seconds of the struggle phase. One diver had a struggle phase that lasted only 1 minute and had no value entered in the late struggle category. Below the plethysmogram is the Hilbert transform showing a progression to larger peaks late. The fourth trace is a plot of the RR intervals. The RR interval variance increases late. The last trace, the standard deviation of RR interval durations (known as the SDNN) (in 8‐interval segments), also shows the increased interval variance. Units for the signals are shown on the plot

**FIGURE 2:**
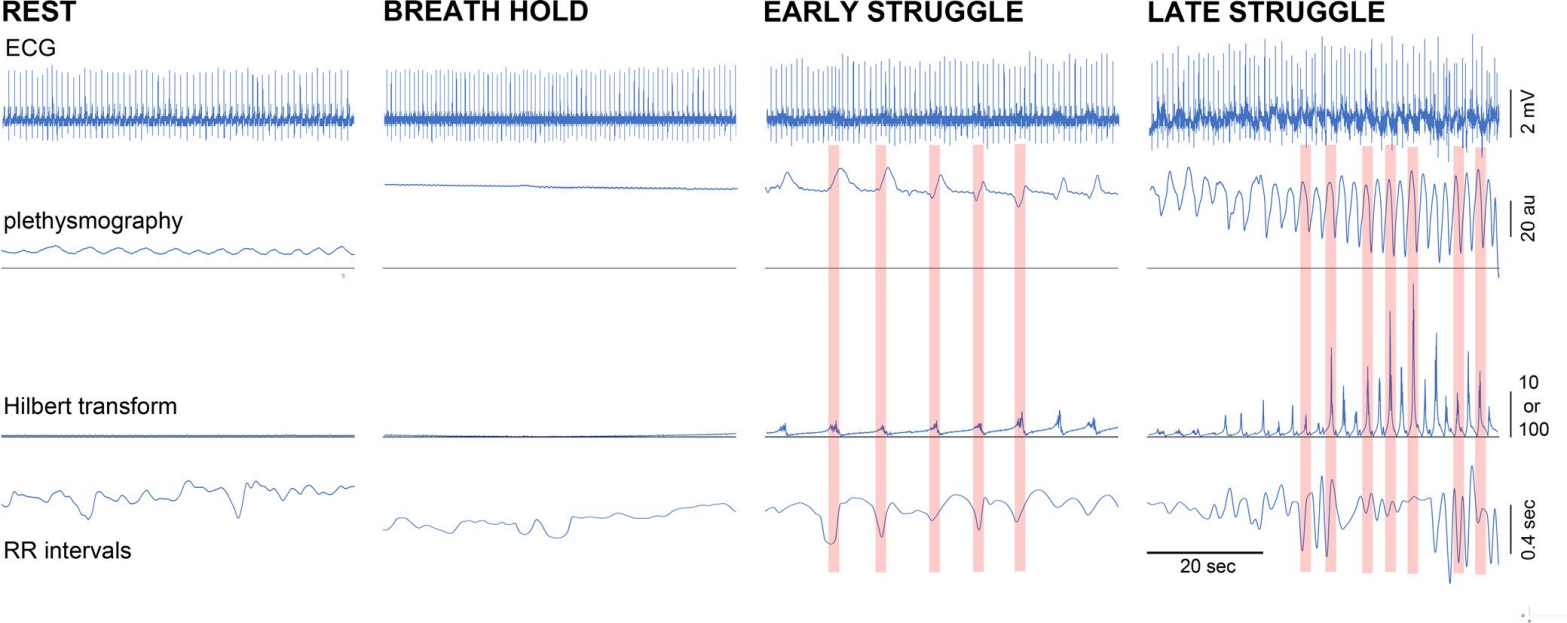
Segments of the breath hold and resting breathing to illustrate features in greater detail. Shown are the ECG, plethysmogram, Hilbert transform, and RR intervals. The plethysmogram and Hilbert transform plots show that EMG bursts are associated with the upstroke of the plethysmogram and show that the respiratory movement‐associated Hilbert signals do not appear during normal breathing or the quiet breath hold phase. Units for the signals are shown on the plot. The scale for the Hilbert transform plots is 10 filtered squared voltage units or 100 filtered squared voltage units for the late struggle phase plot

**FIGURE 3:**
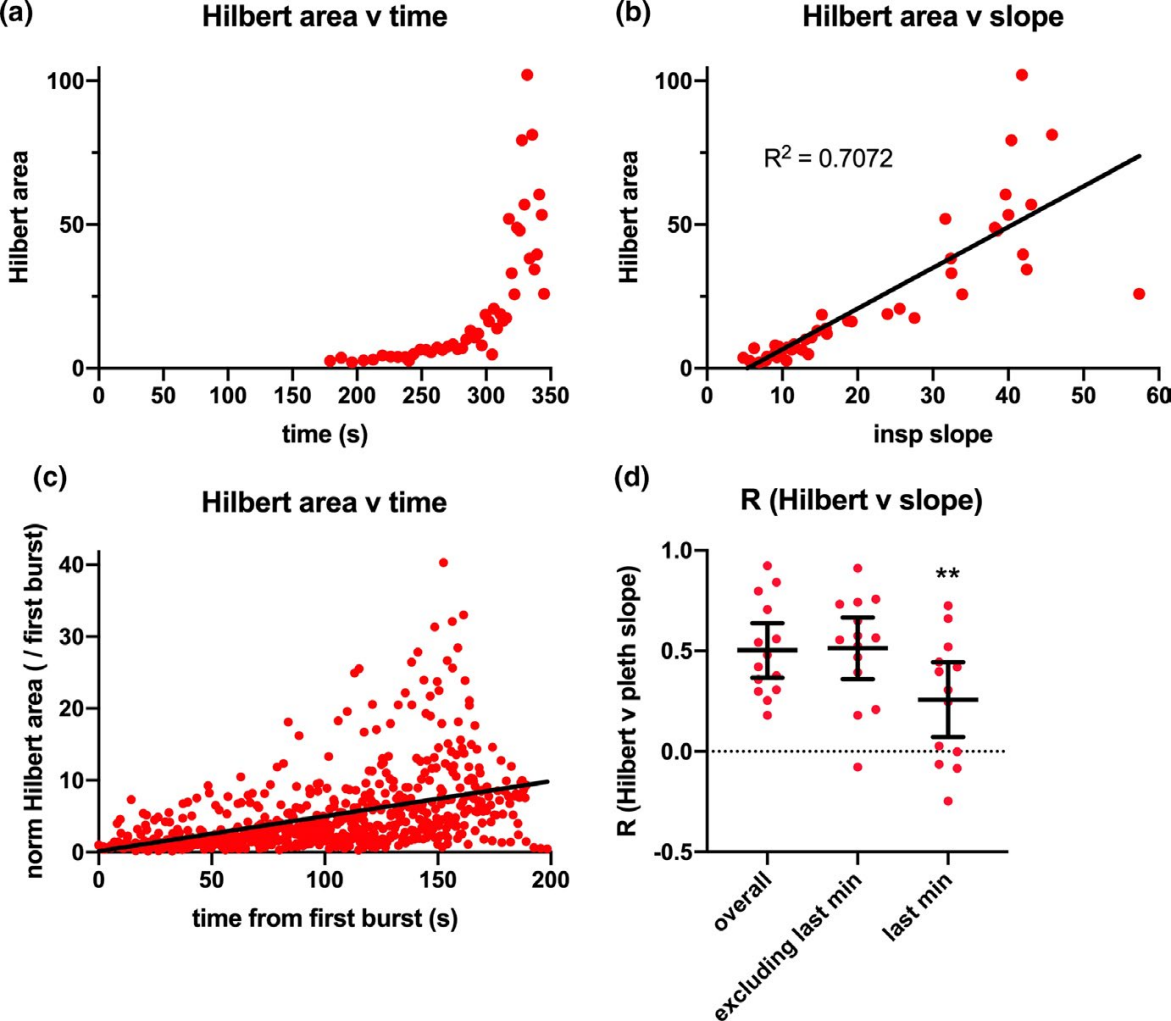
Properties of the extracted EMG signal during breath holds. (a) Example plot of Hilbert area for each peak during the breath hold shows the increase in signal over the transition from early struggle phase to late struggle phase. (b) Plot of Hilbert area against slope of the associated inspiratory phases. Correlations derived from linear regression. (c) Plot of Hilbert area for all subjects (except two––see Methods) aligned in time from the first EMG event marking the onset of the struggle phase. (d) Summary of correlation values for all subjects. Shown are average correlations (with 95% confidence intervals) as labeled. The units for the Hilbert area measure were filtered squared voltage·seconds. The units for the inspiratory attempt slope were arbitrary units from the inductance plethysmogram/seconds. The normalized Hilbert area was calculated by dividing a given Hilbert area by the area of the first burst and this was unitless

EMG bursts in ECG recordings were evident during the struggle phase of breath holds in association with involuntary breath attempts and not during resting breathing (Figures 1 and 2). The average EMG (as quantified by the area under the Hilbert transform envelope of each EMG burst event) increased significantly over the course of the struggle phase (Figure 3a for the individual example shown in Figures 1 and 2) and was 10‐ fold on average higher at the end than at the beginning of the struggle phase (Figure 3c; regression line slope =0.05/s for 12/14 subjects).

From the inductance plethysmograms, we extracted the slopes (calculated from the peak amplitude/time to peak; shown in Figure 3) and peak‐to‐peak amplitudes (data not shown) of inspiratory attempts as indicators of involuntary effort. Slopes and peak‐to‐peak amplitudes were similarly correlated with EMG Hilbert areas and showed similar variance in comparison across divers. The average correlation (R) of Hilbert area with involuntary inspiratory attempt slope (Figure 3d) was 0.5 (range of 0.2–0.9). The correlation between EMG and plethysmogram slope degrades late in the breath hold, presumably due to the mixing of increasing voluntary effort with the involuntary breath attempts [Figure 3d; F(1.185, 14.81) =7.231; *p* = 0.0084; *p* = 0.0028, overall correlation vs correlation for only the last minute; means were 0.50, 0.51, and 0.26, respectively].

### 3.3 Other cardiorespiratory features of prolonged breath holds

Cardiovascular and respiratory effort are known to vary during periods of central apnea (which resembles the quiet phase of a breath hold) and obstructive apnea (which resembles the struggle phase of a breath hold).

**FIGURE 4:**
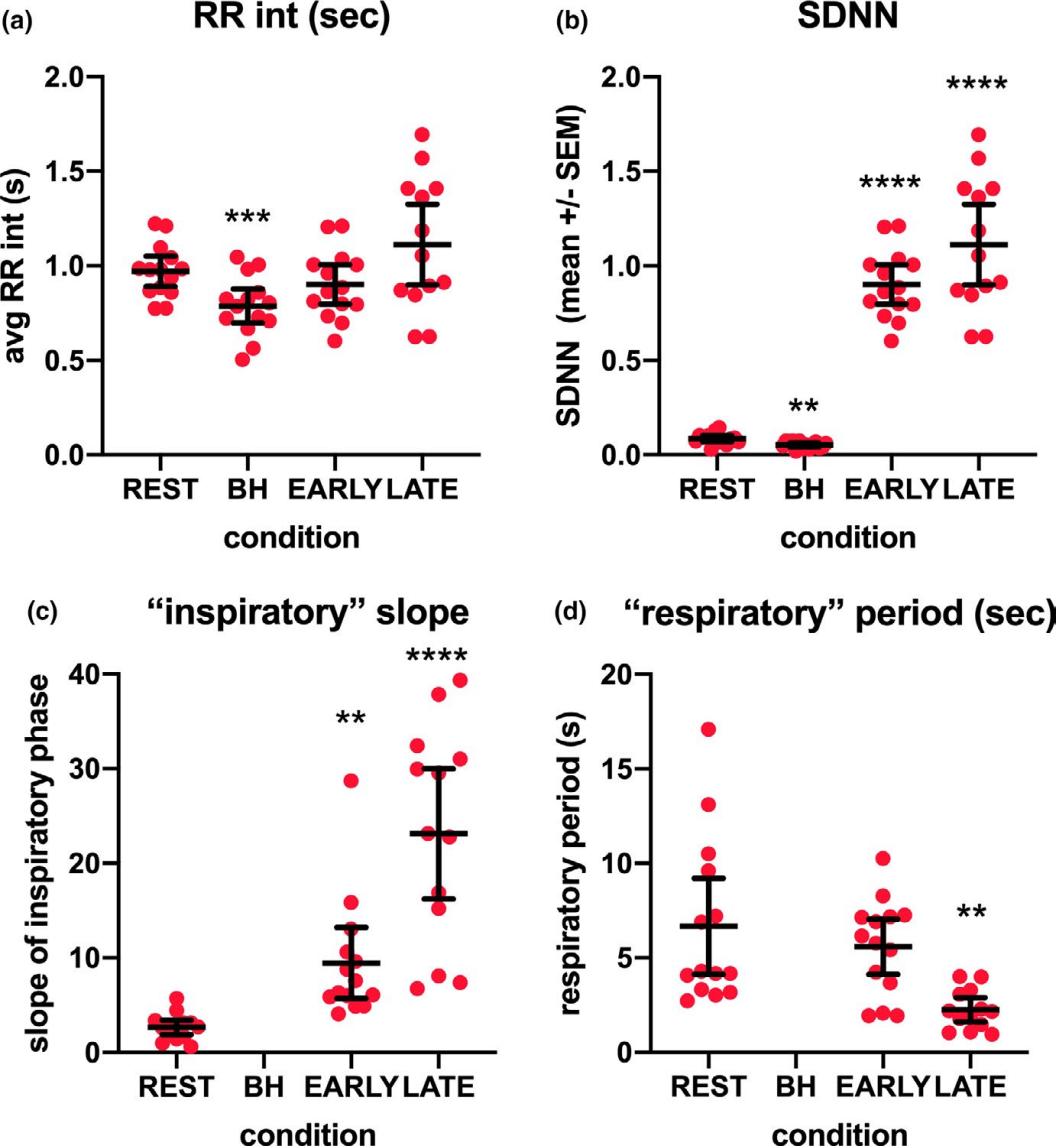
Cardiac and respiratory measures during breath holds. (a) Mean RR interval (seconds) for each 60 second segment across all subjects. Means with 95% confidence intervals are shown in all panels. (b) SDNN (seconds) during each segment shows an initial decrease (due to the elimination of respiratory modulation of RR interval duration) and increases in the early and late struggle phases. (c) Slope of involuntary inspiratory attempts (arbitrary units / seconds) shows significant increases in the early and late struggle phases. (d) The interval between involuntary breath attempts (“respiratory” period in seconds) is significantly shorter during the late struggle phase. As breath attempt frequency is the reciprocal of the breath attempt period, a shorter period corresponds to a higher frequency

RR intervals: RR intervals were shorter in the last minute of the quiet breath hold compared to rest and had less variance (Figures 2 and 4). This can be attributed to the absence of respiration‐associated intrathoracic pressure changes that alter cardiac filling times. For the RR intervals (Figure 4a), F(1.383, 17.52) =8.725; *p* = 0.0050; *p* = 0.0006 for rest versus breath hold after post hoc correction. For SDNN (Figure 4b), F(1.276, 16.16) =143.7; *p* < 0.0001; *p* = 0.001 for rest versus breath hold and *p* < 0.0001 for rest versus early and late struggle phases. RR interval variance increased significantly in the struggle phase and showed minima in association with involuntary inspiratory attempts.

Involuntary respiratory attempts: We measured the slope of the inspiratory phase of involuntary breath attempts, the peak‐to‐peak amplitudes, and event frequency. Figure 4c shows that the inspiratory slope is significantly steep during struggle phase inspiratory attempts (consistent with increased inspiratory attempt effort) compared to rest [F(1.574, 19.67) =35.11; *p* < 0.0001; *p* = 0.0037 for rest vs. early struggle and *p* < 0.0001 for rest vs. late struggle]. Figure 4d shows the increase in breath attempt frequency as a decrease in the respiratory period [F(1.164, 22.11) =7.740; *p* = 0.0084; *p* = 0.0067 for rest vs. late struggle period]. Not illustrated for all participants (but exampled in Figures 1 and 2) is the change in peak‐to‐peak amplitude of involuntary breaths, which were significantly larger in the late vs. onset of the struggle phase [F(1.707, 21.33) =10.32; *p* = 0.0011; *p* = 0.0050 for rest vs. late struggle].

## 4 DISCUSSION

We analyzed broad bandpass ECG recordings taken from experienced breath holding divers while they performed prolonged breath holds in a resting and dry laboratory setting. We extracted a quantitatively large EMG signal from ECG recordings and showed a correlation of this EMG signal to measures of involuntary inspiration attempts (notably the slope of the inductance plethysmogram for the initial phase of the inspiratory attempt) that characterize the struggle phase of the breath hold. Upon quantification with Hilbert transforms, we found that there was a magnitude increase in EMG bursts over the course of the struggle phase that correlated better with the slope of the inductance plethysmogram inspiratory phase than the peak‐to‐peak plethysmogram amplitudes. An increase in heart rate variability and a shift of RR interval minima from expiration at rest (in normal HRV analyses, RR interval minima coincide with expiration and RR interval maxima coincide with inspiration) to inspiration during the struggle phase (i.e., the relation between RR interval minima and maxima is reversed so that RR interval minima coincide with inspiration and RR interval maxima coincide with expiration) are similar to the features of obstructive apnea (Stewart et al., 2017). Our findings emphasize the fact that the EMG signal extracted from the ECG can complement inductance plethysmography during breath holds or obstructive apnea and may even be a better indicator of involuntary effort than plethysmography (the current “gold standard”). This conclusion is based on our finding that measures of the EMG associated with inspiration attempts reveal the progressive inspiratory effort better than inductance plethysmography. Our results demonstrate that the inspiratory effort‐associated EMG signal can complement plethysmography or used by itself when plethysmography is not available. Moreover, we suggest that the struggle phase of a prolonged breath hold can be used to partially simulate periods of pathological apnea for studies in OSA and epilepsy. The value of this point is that controlled monitoring of breath holds can be performed where such monitoring is more difficult during sleep and even more difficult during seizures where apneic periods are quite rare.

### 4.1 Breath hold phases and relation to apnea in other contexts

The absence of breathing during the quiet easy phase of the breath hold is not due to a voluntary suppression of the central respiratory rhythm, but a voluntary suppression of respiratory motor behavior (Parkes, 2006). The easy phase can be extended when the apnea is accompanied by a powerful dive response that conserves and prioritizes oxygen‐rich blood to the central organs (brain and heart) (Panneton, 2013; Panneton & Gan, 2020). In this respect it may be comparable to the unconscious/involuntary apnea seen in association with seizure activity that occurs together with modest bradycardia, an open airway, and no involuntary breathing movements (Lacuey et al., 2017; Mooney et al., 2019; Nakase et al., 2016).

The struggle phase is different, as the voluntary suppression of respiratory motor behavior is won over by powerful involuntary contractions of the respiratory muscles. The increase in EMG amplitude as the struggle phase progresses bears a striking resemblance to what has been observed in obstructive apnea under various conditions, including obstructive sleep apnea (Berry et al., ,,^2016^, ^2019^), obstructive apnea associated with epileptic seizure activity in people (Lacuey et al., 2018; Sivathamboo et al., 2020) and in experimental animals (Irizarry et al., 2020; Jefferys et al., 2019; Nakase et al., 2016). This close resemblance is the reason that we suggest that the struggle phase of a prolonged breath hold can be used as a model for obstructive apnea found during sleep or in association with epileptic seizures conditions where the periods of obstructive apnea are unpredictable and where their durations can be much shorter (sleep) or life threatening (epilepsy). In the epilepsy context, it is the acute impact of the apneic episode, more than a cumulative autonomic derangement, that is critical for sudden death in epilepsy (SUDEP) (Stewart et al., 2020), the most significant cause of death in persons with epilepsy (Barot & Nei, 2019; Ryvlin et al., 2013).

EMG bursts in ECG recordings occur in relation to extremes in effort and significant EMG signals do not appear in relation to resting breathing, even during attempts to specifically record them (Jensen et al., 2017). The similarities in EMG activity between voluntary breath holds and clinical obstructive apnea exist despite the differences in lung volumes and partially divergent swings in pleural pressures in obstructive apnea versus breath hold involuntary breathing movements. Indeed, an entirely unique attribute of the breath hold struggle phase is that the involuntary breathing movements manifest in a Mueller maneuver (negative swing in pleural pressure) followed by a Valsalva maneuver (positive swing in pleural pressure) as a consequence of holding the breath at or near total lung capacity (Heusser et al., 2009; Palada et al., 2008). Conversely, obstructive apnea manifests in a Mueller maneuver only, with lung volumes at functional residual capacity.

The similarities of the struggle phase of the breath hold to obstructive apnea go beyond the EMG. Changes in RR interval variation illustrated in Figure 4 and the reversal of the relationship of RR interval minima from expiration to inspiration highlight the associated biomechanics that are similar. This “inverse” relation of short RR intervals to inspiration (i.e., short intervals associated with inspiration) has been described previously in relation to breath holds (Cooper et al., 2004; Parkes, 2006) and controlled airway occlusion experiments (Stewart et al., 2017). The direction of the changes in RR interval are the same between the breath holds and obstructive apnea, but the interval extremes are larger in obstructive apnea due to the greater prevalence of missed beats and the occurrence of doublets and triplets in the ECG (Stewart et al., 2017). Other similarities include increases in blood pressure and sympathetic nerve activity (Heusser et al., 2009; Narkiewicz & Somers, 2003; Narkiewicz et al., 2005; Zbrozyna & Westwood, 1992).

Interestingly, the frequency of involuntary inspiratory attempts increases in the breath holding dives whereas this frequency tends to decrease in obstructive apnea that is free from a subject's attempts to control the involuntary behavior. Paradoxically, the frequency of inspiratory attempts may initially help prolong the voluntary breath holding time, as transient Mueller and Valsalva maneuvers are known to relieve, in part, the multifaceted stress of breath holding (Bain et al., 2018; Bartlett, 1977) and help restore cerebral oxygenation (Dujic et al., 2009; Palada et al., 2008). Exactly why the frequency of involuntary inspiratory attempts differs with obstructive versus voluntary apnea is unclear, but it is more than likely involves conscious control during breath holds that does not occur during obstructive sleep apnea.

### 4.2 Broader utility of a voluntary obstructive apnea model

Why does it matter that the struggle phase might be considered a period of voluntary obstructive apnea? Studies of the physiology associated with obstructive apnea of sleep or associated with seizure activity are limited by the availability of data that can be obtained when such events are captured. Although, many apneic events can be captured in a single sleep episode in an individual diagnosed with severe OSA, event durations impact the physiological consequences. Capturing near miss sudden death events in epileptic patients is considerably more difficult. A “model” of obstructive apnea that can be studied under controlled conditions for minutes would be an invaluable resource for studies of mechanism and intervention.

In the late struggle phase, severe muscular discomfort associated with involuntary inspiratory attempts is often cited by elite apneists as the cause for breaking the breath hold. This discomfort likely parallels the increased EMG activity (Cross et al., 2013). Insights from the role of lung volumes (e.g., starting a breath hold at functional residual capacity) and effort/metabolism on the rate of desaturation will inform the clinical challenges of obstructive sleep apnea and seizure‐associated obstructive apnea, a condition that may underlie sudden death in epilepsy (Stewart et al., 2020).

Another consideration is, paradoxically, whether the training used by experienced divers may offer some benefit to patients at risk for obstructive apnea as a preventative measure to improve tolerance of obstructive events and thus increase their resistance to acute catastrophic outcomes. For example, when resting muscle sympathetic nerve activity and blood pressure are examined in breath holding divers and compared to control subjects, no differences are observed, indicating that experienced divers are not apparently experiencing the cumulative effects of intermittent hypoxia that one might have expected (Breskovic et al., 2010). These findings are corroborated by the fact that lifelong breath hold divers, the Ama divers of Japan, have lower arterial stiffness and better vascular function compared to their healthy non‐diving peers living in the same village (Sugawara et al., 2018; Tanaka et al., 2016). Moreover, trained breath hold divers (Ivancev et al., 2007, 2009) seemingly do not experience the cerebrovascular problems that have been associated with chronic OSA (Dempsey et al., 2014; Durgan & Bryan, 2012). There must be a unique but undefined facet of volitional apnea (e.g., lung volumes or autonomic control) that makes it “healthier” compared to clinical forms of apnea. Future study using simple ECG derived EMG signals may help inform the unique components of volitional apnea that might be beneficial to harness as a therapeutic or training tool in OSA and epilepsy. Importantly, breath training of other types, such as singing (Courtney, 2020; van der Weijden et al., 2020) and restrictive breathing (Kido et al., 2013) has been proposed to help patients with OSA.

## 5 CONCLUSION

With EMG signals extracted from simple lead II ECG, we have identified similarities between the struggle phase of prolonged breath holds and clinical obstructive apnea. We have also highlighted differences that may serve to better understand the pathological consequences of clinical obstructive apnea versus volitional apnea. Finally, the struggle phase of the breath hold defined by EMG signals could serve as a useful model of OSA because event durations can be longer, the timing of events is controlled, subjects are conscious and can describe experiences, and subjects can be manipulated or monitored in ways that are difficult without disrupting sleep.

## CONFLICT OF INTEREST

Neither author has financial interests or connections that might be perceived as a conflict of interest.

## AUTHOR CONTRIBUTIONS

Mark Stewart ‐ Conceptualization, Methodology (signal processing), Formal Analysis, Writing––Original Draft, Writing Review and Editing. Anthony Bain ‐ Conceptualization, Methodology (collecting data from divers), Formal Analysis, Writing—Original Draft, Writing Review and Editing.
